# Supplementary material for: Insights from a Computational-Based Approach for Analyzing Autophagy Genes across Human Cancers
Source: Genes (Basel). 2023 Jul 28;14(8):1550. doi: 10.3390/genes14081550 (PMC10454514; doi:10.3390/genes14081550)
Supplement: Supplementary file 1 [file genes-14-01550-s001.zip › genes-2509751-supplementary figures.pdf]

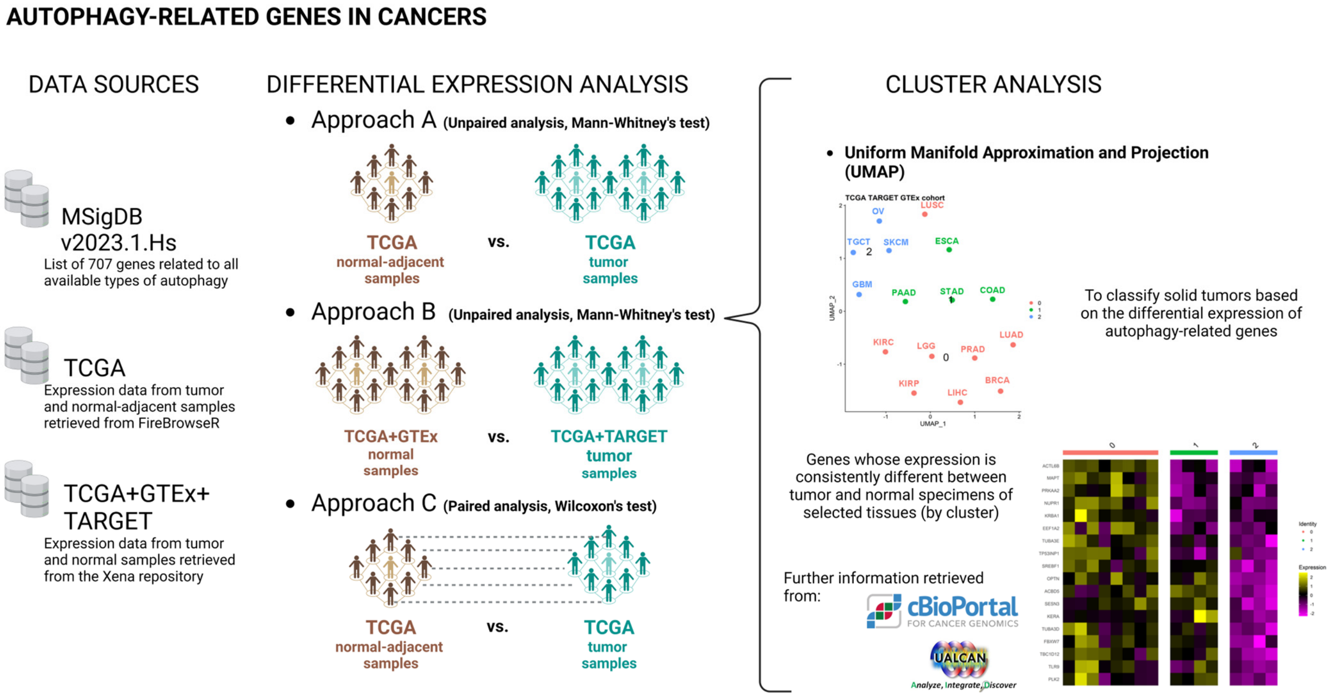

Figure S1: workflow used in this study.

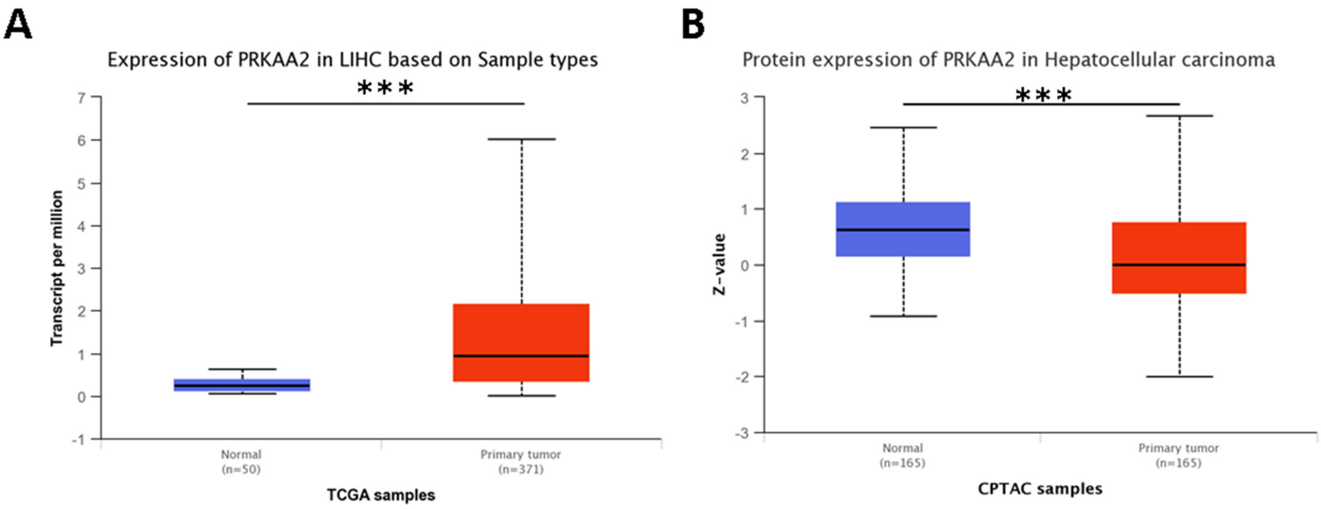

Figure S2: *PRKKA2* is an autophagy-related gene with divergences in its transcript and proteomic levels in LIHC. We evaluated transcriptomic (A) and proteomic (B) levels of the *PRKKA2* gene in the LIHC dataset. Results are contrasting. *** represents comparisons with p value < 0.001 on Welch's t-test.

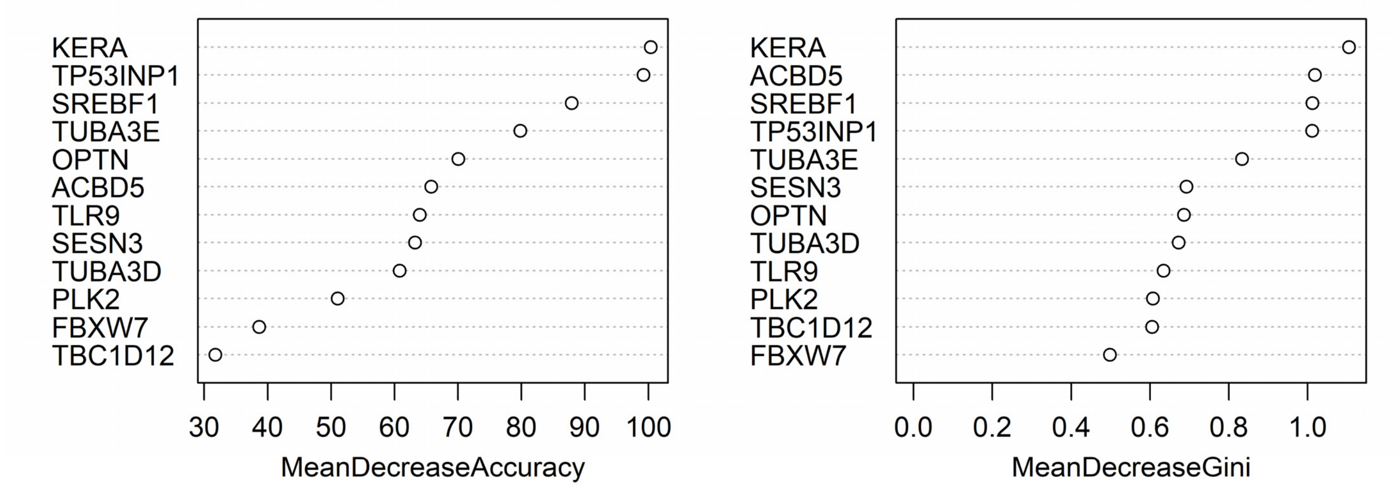

Figure S3: accuracy (left) and Gini (right) contributions of genes relative to the clusters 0 + 1 vs. 2.
